# Ascorbate peroxidase overexpression protects *Leishmania braziliensis* against trivalent antimony effects

**DOI:** 10.1590/0074-02760180377

**Published:** 2018-11-14

**Authors:** Douglas de Souza Moreira, Mariana Vieira Xavier, Silvane Maria Fonseca Murta/

**Affiliations:** 1 Fundação Oswaldo Cruz-Fiocruz, Instituto René Rachou, Belo Horizonte, MG, Brasil

**Keywords:** Leishmania braziliensis, ascorbate peroxidase, antimony resistance, oxidative defence

## Abstract

Ascorbate peroxidase (APX) is a redox enzyme of the trypanothione pathway that converts hydrogen peroxide (H_2_O_2_) into water molecules. In the present study, the APX gene was overexpressed in *Leishmania braziliensis* to investigate its contribution to the trivalent antimony (Sb^III^)-resistance phenotype. Western blot results demonstrated that APX-overexpressing parasites had higher APX protein levels in comparison with the wild-type line (LbWTS). APX-overexpressing clones showed an 8-fold increase in the antimony-resistance index over the parental line. In addition, our results indicated that these clones were approximately 1.8-fold more tolerant to H_2_O_2_ than the LbWTS line, suggesting that the APX enzyme plays an important role in the defence against oxidative stress. Susceptibility tests revealed that APX-overexpressing *L. braziliensis* lines were more resistant to isoniazid, an antibacterial agent that interacts with APX. Interestingly, this compound enhanced the anti-leishmanial Sb^III^ effect, indicating that this combination represents a good strategy for leishmaniasis chemotherapy. Our data demonstrate that APX enzyme is involved in the development of *L. braziliensis* antimony-resistance phenotype and may be an attractive therapeutic target in the design of new strategies for leishmaniasis treatment.

Leishmaniasis is an important neglected tropical disease caused by different species of unicellular protozoan parasites belonging to the *Leishmania* genus. The three main clinical manifestations of this illness are cutaneous (CL), mucocutaneous (MCL) and visceral (VL).^1^ *Leishmania (Viannia) braziliensis*, which is broadly distributed in the Americas, is the aetiological agent of both CL and MCL.^2^ It is estimated that 700,000 to one million new cases of leishmaniasis and 20,000 to 30,000 deaths occur annually.^3^

Chemotherapy is the main form of disease control, since there is no human vaccine available for use.^4^ Thus, pentavalent antimony (Sb^V^)-based compounds (meglumine antimoniate - Glucantime^®^, and sodium stibogluconate - Pentostam^®^) for several decades have been the principal drugs employed to treat all disease forms in many countries.^5^ Nevertheless, the mode of antimony action has not been completely elucidated. It is accepted that Sb^V^ is a prodrug that is reduced to the trivalent (Sb^III^) form, which has leishmanicidal effects against amastigote and promastigote forms of the parasite.^6^ Some studies have indicated that Sb^V^ inhibits glycolysis and fatty acid oxidation.^7^

Many cases of antimony resistance have been reported in different countries, especially in Bihar (India), where the treatment failure rates for antimonials reached 65%.^8^ Several mechanisms of resistance to these drugs have been proposed in the literature, such as a lower rate of drug reduction/activation, a decreased uptake or an increased efflux/sequestration of active molecules, gene amplification and higher activity of repair mechanisms due to the drug-induced damage.^9^

Ascorbate peroxidase (APX) is a redox enzyme of the trypanothione pathway that converts hydrogen peroxide (H_2_O_2_) into water molecules, thus regulating oxidative stress in *Leishmania* and avoiding damage to the parasite cells.^10^ Previous studies demonstrated that *Trypanosoma cruzi* extracts contained ascorbate-dependent peroxidase activity.^11^ ^,^ ^12^ ^,^ ^13^ Nogueira et al.^14^ showed that the APX level was increased in benznidazole-resistant *T. cruzi* populations. APX is an important factor that controls metacyclogenesis and apoptosis in *L. major*.^15^ Interestingly, Mukherjee et al.^16^ demonstrated an intra-chromosomal amplification of a sub-telomeric locus on chromosome 34, a region coding for APX, in antimony-resistant *L. major*. Since APX is absent in humans and it presents an important role in the antioxidant defence of the trypanosomatids, this enzyme may be considered an excellent drug target for chemotherapy of these parasites.^17^

Considering a variety of resistance mechanisms to antimonials in *Leishmania*, it has become necessary to discover new targets to develop other therapeutic strategies to control the disease. Thus, the aim of this work was to overexpress the APX gene in *L. braziliensis* to investigate the contribution of this enzyme to the antimony-resistance phenotype of this parasite.

Promastigote forms of *L. braziliensis* (MHOM/BR/75/M2904) were grown at 26ºC in M199 medium supplemented as previously described.^18^ All analyses were performed with parasites in the exponential growth phase.

To generate APX-overexpressing lines, a 918-bp fragment corresponding to the APX-coding region (TriTrypDB accession number LbrM.20.0150) was amplified with *Pfx* DNA polymerase (Invitrogen) from *L. braziliensis* genomic DNA using the forward primer:5’-TGGATCCCCACCATGACCGGTACCTCGCGG-3’ and the reverse primer: 5’-TTGGATCCTTAGCATTCCACTGCCGGTG-3’. The underlined sequences correspond to the *Bam*HI restriction site. The next steps were performed as previously reported.^19^ Briefly, the APX amplicons were cloned into the pGEM-T Easy^®^ vector (Promega, Madison, WI, USA), digested with *Bam*HI enzyme and introduced into the dephosphorylated pIR1BSD expression vector (kindly provided by Dr Stephen Beverley, Washington University, USA). After that, the pIR1BSD (empty vector) and pIR1BSD-APX constructs were linearised by *Swa*I digestion, electroporated into wild-type *L. braziliensis*, and the colonies were obtained on semisolid M199 medium containing 10 µg/mL blasticidin (BSD). After two weeks, genomic DNA from transfected clonal lines was subjected to PCR tests with primers specific for the BSD marker that confers resistance to blasticidin. The results indicated the presence of a 399-bp fragment in all blasticidin-resistant clones (data not shown), confirming successful transfection.

Western blot assays were carried out to test whether the clones overexpressed APX protein. Protein extracts from parasites were obtained according to the protocol previously described.^19^ Total proteins (20 µg) were separated by electrophoresis on 12% SDS-polyacrylamide gel, electrotransferred onto nitrocellulose membrane (Bio-Rad, Hercules, CA, USA), blocked, washed and probed with rabbit polyclonal *T. cruzi* anti-APX antibody (1:20)^14^, for 12 h at 4ºC in blocking solution. The blots were washed and incubated with horseradish peroxidase-conjugated anti-rabbit IgG antibody (1:1,000) (GE Healthcare), washed, exposed to ECL Plus chemiluminescent substrate (GE Healthcare) and revealed by ImageQuant LAS 4000 (GE Healthcare). It is important to note that the APX amino acid sequences of *L. braziliensis* and *T. cruzi* had 63% identity (data not shown). Western blot results showed that in all *Leishmania* samples evaluated, the *T. cruzi* anti-APX antibody recognized a 34 kDa polypeptide, corresponding to the expected size of APX protein (Fig. 1A). Normalisation of the results with the monoclonal anti-α-tubulin antibody (1:15,000) (Sigma, St. Louis, USA) revealed that the APX protein level was 5-fold higher in transfected clones 4 and 13 from *L. braziliensis* than in the wild-type or transfected with empty vector (controls) (Fig. 1A).

Promastigotes of wild-type *L. braziliensis* and APX-overexpressing cell lines were incubated in M199 medium at 2 x 10^6^ cells/mL in 24-well plates in the absence or presence of increasing concentrations (0.6-149.7 μM) of potassium antimonyl tartrate (Sb^III^) (Sigma-Aldrich, St. Louis, MO, USA) for 48 h. The effective concentration required to decrease the growth by 50% (EC_50_) was determined using a model Z1 Coulter Counter (Beckman Coulter, Fullerton, CA, USA). The EC_50_ values were obtained from three independent measurements in triplicate, using the linear interpolation method.^19^ The data indicated that the Sb^III^ EC_50_ of the untransfected *L. braziliensis* line (LbWTS) was 7.3 μM, whereas clones 4 and 13 had EC_50_ values of 58 μM and 55.6 μM, respectively (Fig. 1B). This result demonstrates that these clones were approximately 8-fold more resistant to trivalent antimony in comparison with the untransfected control (LbWTS).

APX-overexpressing *L. braziliensis* clones were also subjected to susceptibility assays with H_2_O_2_ to analyse their tolerance to oxidative stress produced by several concentrations (100-400 μM) of this compound during a 48 h incubation. Our results revealed that the LbWTS line had an H_2_O_2_ EC_50_ of 184 μM, whereas LbAPX clones 4 and 13 had EC_50_ values of 324 μM and 304 μM, respectively (Fig. 2A). These data indicate that the resistance index for these clones was approximately 1.8-fold higher than for the wild-type line, suggesting that APX enzyme plays an important role in the defence against oxidative stress in *L. braziliensis*.

The amino acid sequence of APX was used for a search of possible drugs against this enzyme in DrugBanK (www.drugbank.ca), which returned the antibacterial agent isoniazid (DB00951). This drug, a synthetic derivative of isonicotinic acid, is used in the treatment of tuberculosis. Isoniazid interferes with the mycolate-synthetase enzyme, which is important in the synthesis of mycolic acid, a fundamental component of the mycobacteria cell wall.^20^ Other mechanisms of action have been uncovered, such as chelation of metallic ions necessary for mycobacterial metabolism and interference in glucose metabolism and cellular respiration of mycobacteria.^21^ Another study reported the first crystal structures of isoniazid complexes with APX.^22^ Isoniazid is also part of the drug cocktail used by HIV-positive patients. It is used as a prophylaxis against tuberculosis, since these patients are frequently exposed to *M. tuberculosis* and their immune systems are deficient.^23^ Thus, we incubated the wild-type *L. braziliensis* line and parasites transfected with the pIR1BSD-APX construct with increasing concentrations of isoniazid (200-10,000 μM) for 48 h and determined the isoniazid EC_50_ for these parasites. Clones 4 and 13 overexpressing APX had EC_50_ values of 838 and 707 μM, respectively (Fig. 2B). These values were 1.5- and 1.3-fold higher in comparison with the LbWTS line, which presented an isoniazid EC_50_ of 563 μM, demonstrating that both clones were more resistant to this drug. This result shows that overexpression of APX enzyme protects parasites from a lethal effect of the inhibitor isoniazid. Furthermore, the isoniazid effect on the growth of *L. braziliensis* lines exposed to Sb^III^ was also evaluated in this study. Interestingly, the combination of these two drugs increased the leishmanicidal activity against LbWTS and APX-overexpressing clones in comparison to those lines incubated with Sb^III^ or isoniazid alone (Fig. 2C). The anti-leishmanial effect was more pronounced in APX-overexpressing *L. braziliensis* clones 4 and 13, which exhibited a growth inhibition of 82% and 86%, respectively. On the other hand, the growth inhibition in the LbWTS line was 73%. These data indicate that the combination of Sb^III^ and isoniazid produced a higher lethal effect in parasites overexpressing the APX enzyme, which are more resistant to Sb^III^ and isoniazid, than the wild-type parasites. Therefore, the combination of these two drugs might represent a good strategy to be further evaluated for chemotherapy against leishmaniasis.

**Fig. 1:**
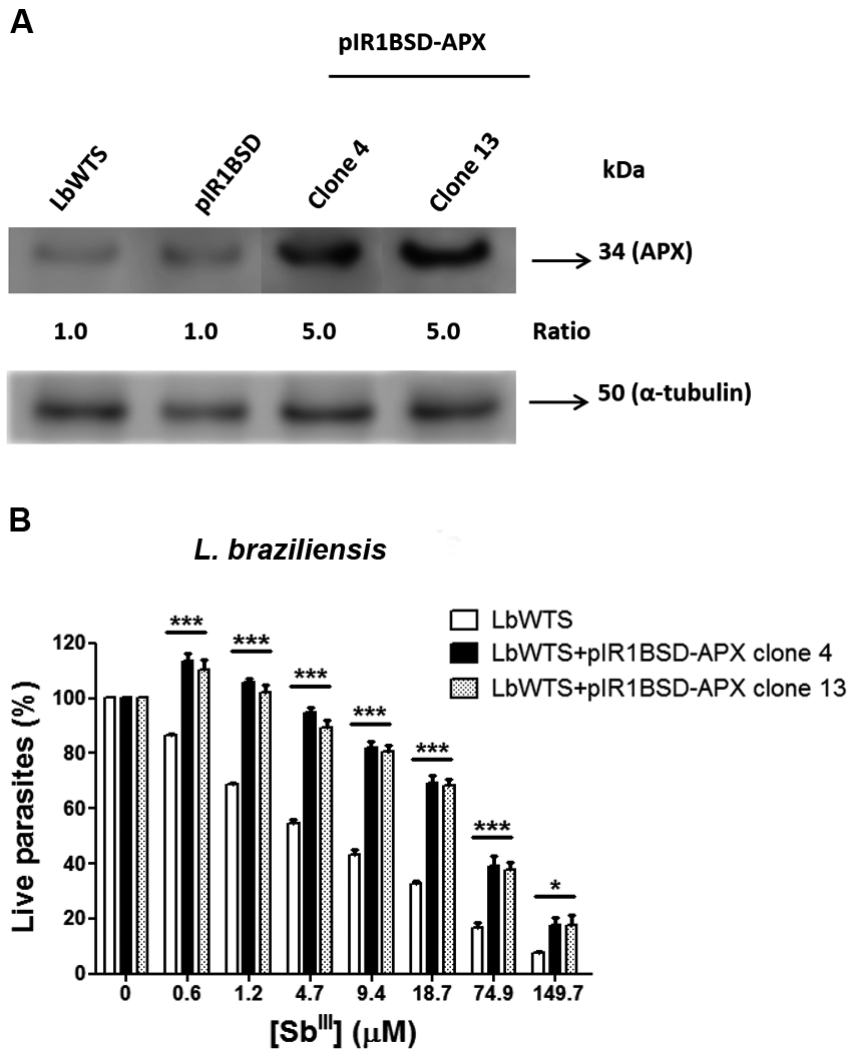
ascorbate peroxidase (APX) protein levels and trivalent antimony (Sb^III^) susceptibility assay of *Leishmania braziliensis* clonal lines untransfected or transfected with the APX gene. (A) Proteins (20 µg) were separated on a 12% sodium dodecyl sulfate-polyacrylamide gel electrophoresis (SDS-PAGE) and blotted onto nitrocellulose membranes. The blots were probed with rabbit polyclonal *Trypanosoma cruzi* anti-APX antibody (1:20) and developed using the ECL Plus kit. The blots were normalised using the anti-α-tubulin monoclonal antibody (1:15,000). The band intensities were quantified using GelAnalyzer 2010 software. The ratio shown is relative to the *L. (V.) braziliensis* wild-type (LbWTS) band (clones/WTS). (B) Parasites were cultured in the absence or presence of increasing Sb^III^ concentrations (0.6 to 149.7 µM) for 48 h, and the percentage of relative growth was determined using a Z1 Coulter Counter. Mean values ± standard deviations from three independent experiments performed in triplicate are indicated. Data were analysed by two-way analysis of variance (ANOVA) followed by a Bonferroni *post hoc* test using GraphPad Prism 5.0 software. Statistically different values are denoted as follows: *p < 0.05; and ***p < 0.001.

**Fig. 2:**
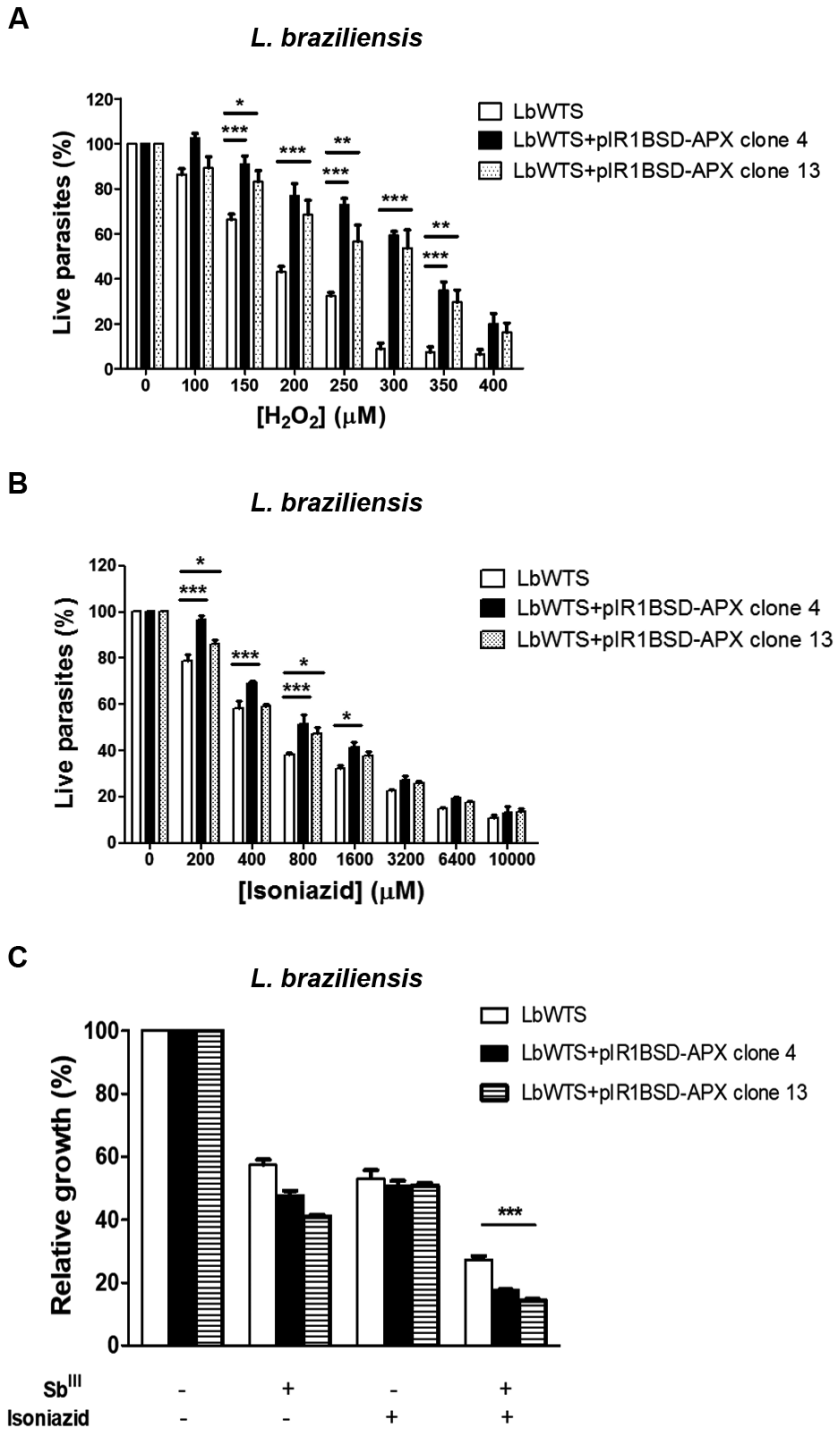
*in vitro* tolerance to exogenous hydrogen peroxide, isoniazid EC_50_ for wild-type and ascorbate peroxidase (APX)-overexpressing *Leishmania braziliensis* lines, and the effect of isoniazid on the growth of *L. braziliensis* lines upon trivalent antimony (Sb^III^) exposure. Parasites were incubated in M199 medium in the absence or presence of different concentrations of (A) H_2_O_2_ (100 to 400 µM) and (B) isoniazid (200 to 10,000 µM). For competition tests (C), cells were exposed to the EC_50_ of Sb^III^ 7.3, 58 and 55.6 μM for the *L. (V.) braziliensis* wild-type (LbWTS) and APX-overexpressing clones 4 and 13, respectively and the EC_50_ of isoniazid (563, 838 and 707 μM for the LbWTS and APX-overexpressing clones 4 and 13, respectively) independently or in combination, followed by incubation for 48 h. The percentage of relative growth was determined using a Z1 Coulter Counter. Mean values ± standard deviations from three independent experiments performed in triplicate are indicated. Data were analysed by one-way or two-way analysis of variance (ANOVA) followed by a Bonferroni *post hoc* test using GraphPad Prism 5.0 software. Statistically different values are denoted as follows: *p < 0.05; **p < 0.01; and ***p < 0.001.

Trypanosomatids are frequently exposed to different reactive oxygen species (ROS). These parasites have a peculiar mechanism of antioxidant defence based on the trypanothione reductase system, which maintains an intracellular reducing environment.^17^ This defence machinery is composed of many enzymes distributed in diverse cellular compartments and activated against various oxidants.^24^ APX is a relevant mitochondrial enzyme involved in detoxification of H_2_O_2_ into water molecules. In the present study, transfection of the APX gene in *L. braziliensis* generated clones overexpressing APX protein, as shown by western blot analyses. In addition, functional assays demonstrated that APX overexpression rendered *L. braziliensis* clones 4 and 13 more resistant to Sb^III^. This result indicates that greater amounts of APX enzyme are necessary to reduce the toxic effects produced by the drug and to prevent parasite death due to perturbations to its redox potential. Thus, Wyllie et al.^25^ suggested that Sb^III^ causes alterations in the thiol redox potential of *Leishmania*, which can lead to cell death by oxidative stress. Interestingly, APX overexpression in *L. major* provoked the depletion of mitochondrial ROS burden and resistance to cardiolipin oxidation.^26^ Kumar et al.^10^ showed that APX overexpression in the amphotericin B-resistant *L. donovani* line rescues cells from the deleterious effect of oxidative stress.

We also investigated whether APX overexpression in *L. braziliensis* protects the parasite from the damage caused by increasing concentrations of exogenous H_2_O_2_. Our results indicated that clonal lines overexpressing the APX enzyme were less susceptible to H_2_O_2_ than the wild-type *L. braziliensis* line. Dolai et al.^26^ demonstrated that overexpression of this enzyme in *L. major* decreased H_2_O_2_-induced lethality, corroborating our data. Additionally, APX overexpression protects this *Leishmania* species against apoptosis induced by oxidative stress generated by H_2_O_2_ or camptothecin treatment.^27^ Interestingly, Pal et al.^15^ showed that deletion of APX in *L. major* renders cells more susceptible to H_2_O_2_. Nogueira et al.^14^ revealed that benznidazole-resistant populations of *T. cruzi* presented higher tolerance to exogenous H_2_O_2_ than their susceptible counterparts. Andrade and Murta^28^ showed that *L. braziliensis* lines overexpressing tryparedoxin peroxidase (TXNPx), an enzyme that is also involved in antioxidant defence, were more tolerant to H_2_O_2_ when compared with the untransfected parental line. These data reinforce the notion that the APX and TXNPx enzymes are needed for detoxifying peroxidase activity, indicating their essential role in the defence against oxidative stress in trypanosomatids.

Isoniazid was found to be a potential inhibitor of APX enzyme during our search in DrugBank. This compound is a bactericidal agent that is active against organisms of the genus *Mycobacterium* and is used to treat all forms of tuberculosis (www.drugbank.ca/drugs/DB00951). A previous study reported that isoniazid can become an inhibitor of peroxidase activity in mutant soybean APX, demonstrating that point mutations in the enzyme active site can contribute to drug resistance.^22^ Our results demonstrated that overexpression of APX enzyme confers resistance to isoniazid. Surprisingly, this drug enhanced the anti-leishmanial effect of Sb^III^, mainly against *L. braziliensis* clones overexpressing APX. This combination of drugs might represent a good strategy to be further elaborated for leishmaniasis chemotherapy. Interestingly, Amorim et al.^29^ showed that pentacyano(isoniazid)ferrate(II), an organometallic compound analogue of isoniazid, is efficient at inhibiting proliferation of *L. braziliensis* promastigote and amastigote forms, suggesting that it is a possible safe drug for treatment of infection caused by this parasite.

This study is the first to show that isoniazid has an effect against *L. braziliensis*. In summary, our study evidences that overexpression of APX enzyme is involved in the mechanism of *L. braziliensis* Sb^III^-resistance. Importantly, earlier studies reported by our research group also indicated that other enzymes, e.g., tryparedoxin peroxidase and iron superoxide dismutase-A, have significant functions in the antioxidant defence and in the maintenance of antimony resistance in *Leishmania*.^28^ ^,^ ^30^ Thus, our data contribute to understanding the participation of APX enzyme in the Sb^III^-resistance mechanism and direct the development of new strategies for leishmaniasis chemotherapy.
